# Nanocarbon synthesis by high-temperature oxidation of nanoparticles

**DOI:** 10.1038/srep24109

**Published:** 2016-04-20

**Authors:** Ken-ichi Nomura, Rajiv K. Kalia, Ying Li, Aiichiro Nakano, Pankaj Rajak, Chunyang Sheng, Kohei Shimamura, Fuyuki Shimojo, Priya Vashishta

**Affiliations:** 1 Collaboratory for Advanced Computing and Simulations, Department of Physics & Astronomy, Department of Computer Science, Department of Chemical Engineering & Materials Science, and Department of Biological Sciences, University of Southern California, Los Angeles, CA 90089-0242, USA; 2 Argonne Leadership Computing Facility, Argonne National Laboratory, Argonne, IL 60439, USA; 3 Department of Physics, Kumamoto University, Kumamoto 860-8555, Japan; 4 Department of Computational Science, Kobe University, Kobe 657-8501, Japan

## Abstract

High-temperature oxidation of silicon-carbide nanoparticles (nSiC) underlies a wide range of technologies from high-power electronic switches for efficient electrical grid and thermal protection of space vehicles to self-healing ceramic nanocomposites. Here, multimillion-atom reactive molecular dynamics simulations validated by *ab initio* quantum molecular dynamics simulations predict unexpected condensation of large graphene flakes during high-temperature oxidation of nSiC. Initial oxidation produces a molten silica shell that acts as an autocatalytic ‘nanoreactor’ by actively transporting oxygen reactants while protecting the nanocarbon product from harsh oxidizing environment. Percolation transition produces porous nanocarbon with fractal geometry, which consists of mostly sp^2^ carbons with pentagonal and heptagonal defects. This work suggests a simple synthetic pathway to high surface-area, low-density nanocarbon with numerous energy, biomedical and mechanical-metamaterial applications, including the reinforcement of self-healing composites.

Silicon carbide (SiC) is a promising material for high-power and high-temperature electronic devices that could significantly improve the efficiency of power switches in electrical grid^1^,^2^. SiC has a large band gap, high breakdown field, and high thermal conductivity. Its ability to form a native oxide layer by thermal oxidation is critical for fabricating metal-oxide-semiconductor (MOS) devices. SiC is also used in thermal protection systems in space vehicles^3^, where controlling high-temperature oxidation of SiC is critical. Recently, oxidation of SiC, especially that of SiC nanoparticles (nSiC), has drawn a great deal of attention both scientifically^4^ and technologically^5^. A remarkable application of nSiC oxidation is autonomous healing of cracks in ceramic matrix composites containing nSiC^5^. Near a crack, nSiC gets oxidized in the high-temperature oxygen environment and forms amorphous silica (SiO_2_), which flows into the damage zone and heals the crack. Self-healing of cracks in brittle ceramics can dramatically increase the reliability and lifetime of structural components and reduce the maintenance cost in a broad range of energy technologies such as turbines for power generation.

The nature of oxidation of nanoparticles, which is essential in these applications, differs dramatically from that of their bulk counterparts^6^,^7^. For example, oxidation of Au-In alloy nanoparticles produces an amorphous Au-rich oxide shell that acts as an active catalyst for CO oxidation reactions^8^. This suggests a viable synthetic route toward stable catalytic nanoparticles. Oxidation of nSiC is expected to differ from the bulk picture presented in the classical work of Deal and Grove^9^.

In this paper, we examine atomistic mechanisms underlying high-temperature oxidation of nSiC and identify unique products in oxidation reactions of nSiC. We have used reactive molecular dynamics (RMD) simulations (see Supplementary Section 1) validated by *ab initio* quantum molecular dynamics (QMD) simulations (see Supplementary Section 2) to study the behavior of nSiC in an oxygen-rich environment. In an RMD simulation, the time evolution of atomic trajectories is determined by an environment-dependent force field based on the concept of reactive bond-order. The reactive force field describes chemical bond breakage and formation, and charge transfer between atoms based on a charge-equilibration approach. In our RMD simulation, a spherical nSiC particle of diameter *D* cut out of 3 C-SiC crystal is embedded in a bath of oxygen molecules (see Methods). We first examine the effect of temperature on the nSiC oxidation process using a nSiC with *D* = 10 nm at various temperatures. Below, we focus on the results at 2,400 K and 2,800 K. Subsequently, we perform simulations of larger nSiC sizes (*D* = 46 and 100 nm) at 2,800 K to study the size effects, with special focus on the formation and morphology of reaction products. The latter simulations have been performed on the 786,432-core IBM Blue Gene/Q computer at the Argonne National Laboratory.

Figure 1a–c, shows snapshots of the simulation for *D* = 10 nm at 2,800 K. Here yellow, cyan, and red spheres represent Si, C, and O atoms, respectively. An animation of the oxidation process is shown in Supplementary movie, S1.mov. Starting from a spherical nSiC immersed in an O_2_ environment (Fig. 1a), initial oxidation produces a silica shell around the unreacted SiC core (Fig. 1b). The overall reaction at high oxygen pressures reads^10^,^11^


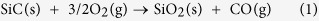


Figure 1b shows the formation of a silica shell on the nSiC surface and release of small oxidized carbon fragments including CO, consistent with Eq. (1). Figure 1b also shows the condensation of graphene-like carbon flakes composed of hexagonal rings in the cavities of the silica shell. The carbon flakes grow into an extended carbon material until the SiC core is completely consumed around 1.7 ns (Fig. 1c). The time evolution of the silica-shell thickness shown in Fig. 1d exhibits a transition from an initial fast oxide growth limited by the reaction rate to slow growth limited by the diffusion of reactants^12^ to the oxide/SiC interface. Although the overall curve is consistent with the linear-to-parabolic transition predicted by the Deal-Grove model^9^, the direct fit to the model is questionable because the shell has large, heterogeneous cavities (Fig. 1c).

Surprisingly, we find the condensation of large graphene-like flakes (colored cyan in Fig. 1b,c) between the unreacted SiC core and silica shell despite in the harsh oxidizing environment at high temperature and high oxygen pressure. Previous RMD simulations of small SiC slabs at similar temperatures indicated incipient graphene-like molecules^11^, which might have been a precursor of the nanocarbon products observed here. Figure 1e,f, shows the number of key chemical bonds as a function of time at temperatures 2,400 and 2,800 K, respectively. The reactants at time 0 consist solely of Si-C and O-O bonds. As expected, these bonds are broken at much higher rates at 2,800 K than at 2,400 K, and a much larger number of Si-O bonds are formed than C-O bonds, reflecting the higher oxidation potential of Si than that of C. Since oxygen reacts more strongly with Si at the silica/SiC interface, carbon atoms are left to form covalent bonds between themselves and condense into nanocarbon products as shown in Fig. 1b,c.

To quantify the growth of graphene-like flakes, we show in Fig. 2a the number of sp^2^-bonded carbon atoms with three carbon neighbors as a function of time at temperatures 2,400 K and 2,800 K for *D* = 10 nm. Larger number of sp^2^ carbons are produced at 2,800 K than at 2,400 K, and a significant fraction of the total number of carbon atoms (~ 2×10^4^) at 2,800 K becomes part of the solid carbon product at the end of the simulation.

To highlight the nature of the nanocarbon product at 2,800 K, Fig. 2b presents a snapshot of only C atoms at 2 ns. The figure shows extended graphene sheets in the system. These are also commonly observed as intermediate products during detonation of energetic materials^13^,^14^. It should be noted that SiC is abundant in the interstellar space, and has been thought of as the source of polycyclic aromatic hydrocarbons that may be a precursor of biomolecules^15^. Interfacial C-rich layers have also been observed during thermal oxidation of SiC in high-power electronic devices^16^,^17^, which is a potential source of poor carrier mobility at SiO_2_/SiC interfaces. QMD simulations suggest that these excess carbons may segregate to form carbon clusters^18^, which is consistent with our observation. Earlier X-ray diffraction measurement also indicated the segregation of graphitic materials in high-temperature SiC crystal at 2,400 K^19^.

To understand the formation mechanism of nanocarbon product and its geometry, we have performed larger simulations with nSiC diameters *D* = 46 and 100 nm. Figure 2c shows snapshots at time 0.2 and 0.4 ns for *D* = 100 nm, where the mass of each carbon cluster in atomic mass unit (amu) is color-coded. At 0.2 ns, a large number of disjoint carbon clusters are formed on the nSiC surface. By 0.4 ns, in contrast, most carbon clusters have been interconnected to form a single nanocarbon that covers the entire surface. Figure 2d shows the size of the largest C cluster as a function of time. We observe a sharp increase in the size of the largest C cluster at 0.34 ns, indicating a percolation transition^20^, in which the entire carbon product is connected. Supplementary movie, S2.mov, animates the percolation transition for carbon clusters during the oxidation of a 100-nm nSiC at 2,800 K. A similar percolation transition was also observed for *D* = 46 nm. Supplementary Fig. S3 shows the size distribution of the graphene flakes (blue) and the size of the largest C cluster (red) as a function of time for *D* = 46 nm.

Percolation transition shown in Fig. 2d is associated with a fractal geometry, which is manifested in the critical cluster-size distribution just before the transition. Figure 2e plots the number of clusters *C*(*i*) as a function of the cluster size *i* at 0.3 ns for *D* = 100 nm. The distribution follows a power law, *C*(*i*) ~ *i*^−*τ*^ for *i* > 10, where the fitted exponent is *τ* = 2.62. According to the theory of percolation, the critical exponent *τ* of the cluster-size distribution is related to the fractal dimension of the clusters as *τ* = *d*/*d*_f_ + 1 (*d* = 3 is the dimensionality)^14^,^21^. The corresponding fractal dimension is *d*_f_ = 1.85. The fractal dimension of aggregates is known to be a sensitive function of growth conditions such as reaction rates and diffusion coefficients^22^. This value thus provides valuable information regarding the reaction mechanisms. This long tail distribution also indicates the abundance of large clusters, and accordingly the need for large-scale simulations. The nanocarbon product is thus a porous material with fractal geometry, which is associated with large internal surface areas and low mass density.

To further characterize the geometry of the nanocarbon product, we calculate the distribution of 5-, 6-, and 7-membered rings formed by C-C bonds. A defect-free graphene sheet would consist of 6-membered hexagons, and 5-membered pentagons and 7-membered heptagons constitute topological defects called disclinations^23^. The positive Gaussian curvature associated with pentagonal defects produces curved surfaces commonly observed in fullerenes^24^, whereas the hyperbolic geometry due to negative-curvature heptagonal defects produces wrinkled surfaces^23^. Figure 3a shows the number of 5-, 6-, and 7-membered rings as a function of time in a 2,800 K RMD simulation for *D* = 10 nm. Most of the C rings are hexagons with approximately equal numbers of pentagonal and heptagonal defects. The inset in Fig. 3a shows the ratios of the numbers of pentagonal and heptagonal defects to the number of hexagons as a function of time. Initially, a large number of pentagons are produced, followed by the production of heptagonal defects, but at the end of the simulation the system has almost the same number of pentagons and heptagons (~17–19%). These topological defects are thought to play a crucial role in tailoring the mechanical^25^ and electronic^26^ properties of graphene. Results in Fig. 3a suggest that it may be possible to control the reaction time and defect densities and thus tune the electronic and mechanical properties of graphene synthesized in nSiC oxidation reaction.

Snapshots in Fig. 3b,c, show the spatial distribution of topological defects in the 2,800 K RMD simulation for *D* = 10 nm at 2 ns. Here, red and blue colors indicate pentagons and heptagons, respectively. We observe a uniform distribution of topological defects in the graphene sheet. Pentagonal and heptagonal disclinations together are known to form a dislocation. The magenta arrow in Fig. 3c indicates alternating 5- and 7-membered rings forming an extensive line defect, *i.e*., a grain boundary, which is commonly observed in graphene sheets^23^. Also shown in Fig. 3c (green arrow) is a Stone-Wales defect^27^, consisting of edge-sharing 5-7-5-7 rings. In Supplementary movie, S3.mov, showing the time evolution of graphene sheets, 5-, 6-, and 7-membered rings are colored red, white, and blue, respectively. Initially, mostly five-membered rings are nucleated, which is consistent with the inset of Fig. 3a. Subsequently, these pentagons act as nucleation seeds for larger graphene sheets composed of mostly 6-membered rings. The initial nucleation of carbon-ring networks with 5-membered rings is consistent with the recently proposed ‘pentagon-first’ mechanism, which is driven by geometry rather than thermodynamic stability^28^. Experimentally observed statistical distribution of 5-membered rings also suggests the non-energetic origin of defect formation^29^.

We observe that graphene flakes nucleate and are ‘woven’ at the nSiC surface, and the silica shell (Fig. 4a) plays a surprisingly active role in the synthesis of graphene flakes. The molten silica shell absorbs environmental oxygen, which becomes part of the Si-O bond network. The O atoms move toward the silica/nSiC interface through a sequence of bond-switching events^30^ and bond preferentially to Si rather than C, as shown in Fig. 1e,f. To quantify oxygen transport in the molten silica shell at 2,400 K and 2,800 K, we calculate the mean square displacement (MSD) averaged over all O atoms bonded to Si (Fig. 4b). Comparison with Fig. 2a shows a positive correlation between the MSD and the amount of carbon product. These results demonstrate that rapid oxygen transport in the molten silica shell plays an essential role in the production of nanocarbon. This autocatalytic role of the silica ‘nanoreactor’^31^ is akin to autocatalytic behavior of reaction products during detonation of pentaerythritol tetranitrate, where H_2_O products are directly involved in the breakage of N-O and formation of C-O bonds^32^. We have also observed such a mechanism in hydrogen production from water by LiAl particles. In that system, QMD simulations reveal that bridging oxygen atoms between Al and Li play an active role in the breaking of O-H and formation of Al-O bonds^33^.

Molten silica is known to catalyze nanocarbon growth^34^,^35^, avoiding the impurity problem of conventional metal catalyzed vapor-liquid-solid (VLS) growth. SiC nanoparticles are also used as catalyzers for chemical vapor deposition (CVD) growth of carbon nanotubes^36^. It has also been suggested that SiC particles act as nucleation centers for interstellar carbon condensation reactions^15^. While being consistent with these earlier works, what is new here is that the silica nanoreactor is self-formed during high-temperature oxidation of nSiC. The molten silica nanoreactor works efficiently, partly because the nanocarbon product is confined in the cavities. A similar confinement effect for efficient discovery of reaction pathways was demonstrated in *ab initio* nanoreactor simulations by Wang *et al*.^31^. Figure 4c–e, shows the geometry of the cavities in the silica shell where the nanocarbon products reside. These cavities create a locally oxygen-deficient environment within which large carbon clusters can grow, protected from the harsh oxidizing environment outside. In summary, the novel fractal geometry of nanocarbon product shown in Fig. 2 arises from topological (*i.e*., pentagonal and heptagonal) defects shown in Fig. 3 and confinement within the silica nanoreactor shown in Fig. 4.

The porous nanocarbon may find numerous energy, biomedical and mechanical-metamaterial applications. Nanocarbon can be extracted from the oxidation product by dissolving silica shells. Potential applications of the nanocarbon with high surface areas and low mass density include supercapacitors^37^ and battery electrodes^38^, and functionalized fractal-graphene grown on luminescent nSiC may also find applications in biomedical sensing^39^. Recently, porous nanocarbon has been suggested as a candidate for a novel “mechanical metamaterial” that could exhibit peculiar mechanical properties such as negative compressibility^40^. Key to the mechanical metamaterial is a low mass density, while maintaining the mechanical integrity. We have estimated the mass density of solid composed of the nanocarbon shown in Fig. 2b to be 0.50 g/cm^3^ (Supplementary Fig. S4). Supplementary movie, S4. mov, shows that this nanocarbon in fact possesses high mechanical integrity during collision with a hard wall. The unique mechanical properties of porous ‘metacarbon’ likely originate from topological defects^41^, which are abundant in the nanocarbon synthesized by nSiC-oxidation as shown in Fig. 3. Furthermore, porous carbon nanoballs shown in Fig. 2b may be assembled into superlubricant^42^. Our results thus suggest a simple synthetic pathway^43^ to porous fractal nanocarbon with broad industrial applications, adding to existing carbide-derived carbon synthesis methods including hydrothermal reaction^44^. Addition of carbon is also known to improve the mechanical strength of ceramic composites^44^, and thus the carbide-derived nanocarbon found in our simulation is expected to reinforce the aforementioned self-healing ceramic nanocomposites^5^. Furthermore, our results provide insight into fundamental scientific issues including carbon condensation in early Earth^45^ and interstellar space^15^, namely, how large carbon products can be formed in harsh environments.

Our first-principles based prediction awaits future experimental tests. After burning nSiC, SiO_2_ can be etched away to isolate the nanocarbon product. The same process was used in recent synthesis of carbon-based supercapacitors^46^. In their work, nanocarbon was grown in mesoporous SiO_2_ using chemical vapor deposition, followed by the etching of SiO_2_ to isolate the carbon product. Our proposed synthetic method is simpler and requires only one step to form a silica nanoreactor and nanocarbon at the same time. Also, carbon production during thermal oxidation of SiC is well documented^16^,^17^, and the proposed nanocarbon synthesis by high-temperature oxidation of nSiC is plausible. Finally, the produced nanocarbon can be characterized by nuclear magnetic resonance (NMR), Raman spectroscopy, and transmission electron microscopy (TEM).

## Methods

To study the size effect, we performed three sets of simulations for nSiC with diameter *D* = 10, 46, and 100 nm. The dimension of the cubic simulation box was 15, 68 and 150 nm for the three cases, and the periodic boundary condition was applied in all Cartesian directions. The total numbers of atoms were 100,195, 10,007,652, and 112,071,581, respectively for *D* = 10, 46 and 100 nm, respectively. In each case, the nSiC surface was first relaxed with the conjugate-gradient (CG) method, and subsequently O_2_ molecules were inserted in the simulation box. A high O_2_ density (1/6th of the SiC mass density) was used to simulate a fuel-lean oxidation condition^11^. The simulations were performed at several temperatures including 2,400 and 2,800 K. In each simulation, the total system including the nSiC and O_2_ molecules was heated up to the desired temperature over 10 ps. Subsequently, uniform temperature distribution was achieved by scaling the atomic velocities during the simulation. The simulations was run for 2 ns until the nSiC was completely oxidized at 2,800 K for *D* = 10 nm, and to the point where the percolation of carbon clusters were confirmed for *D* = 46 and 100 nm. The *D* = 10 nm simulations were performed on a Linux cluster at the University of Southern California, whereas the *D* = 46 and 100 nm simulations were performed on the 786,432-core IBM Blue Gene/Q computer at the Argonne National Laboratory.

## Additional Information

**How to cite this article**: Nomura, K. *et al*. Nanocarbon synthesis by high-temperature oxidation of nanoparticles. *Sci. Rep.* **6**, 24109; doi: 10.1038/srep24109 (2016).

## Supplementary Material

Supplementary Information

Supplementary Movie S1

Supplementary Movie S2

Supplementary Movie S3

Supplementary Movie S4

## Figures and Tables

**Figure 1:**
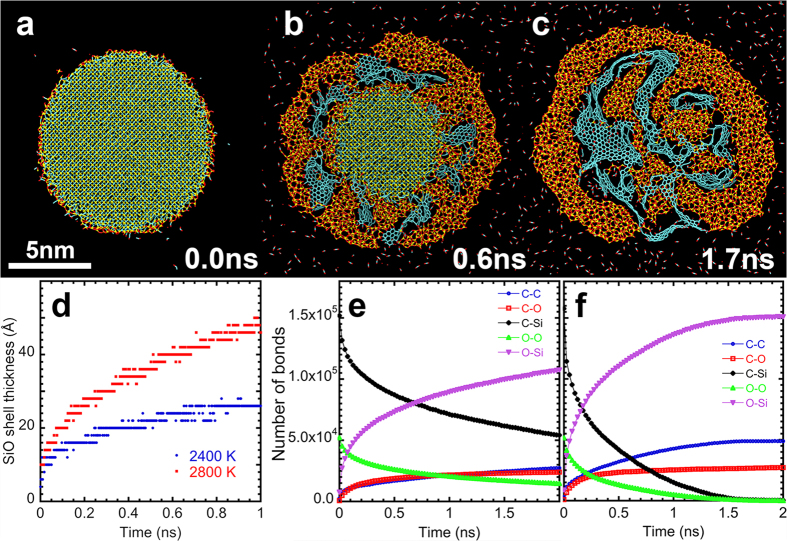
High-temperature nSiC oxidation. (**a–c)** Snapshots from an RMD simulation showing oxidation of a SiC nanoparticle of diameter 10 nm at temperature 2,800 K. A 2 nm-thick slice in the middle of the simulation box is shown in panels (**a**–**c**). Yellow, cyan, and red spheres represent silicon, carbon, and oxygen atoms, respectively, in nSiC. For clarity, O_2_ molecules surrounding nSiC are not shown here. (**a**) Initial configuration; (**b**) a porous layer of silica encapsulating carbon products develops after 0.6 ns; and (**c**) carbon clusters grow further until the core of nSiC is completely oxidized around 1.7 ns. (**d**) The time evolution of the silica-shell thickness at temperatures 2,400 K (blue) and 2,800 K (red). (**e**,**f**) The time evolution of the number of chemical bonds at 2,400 K (**e**) and 2,800 K (**f**).

**Figure 2:**
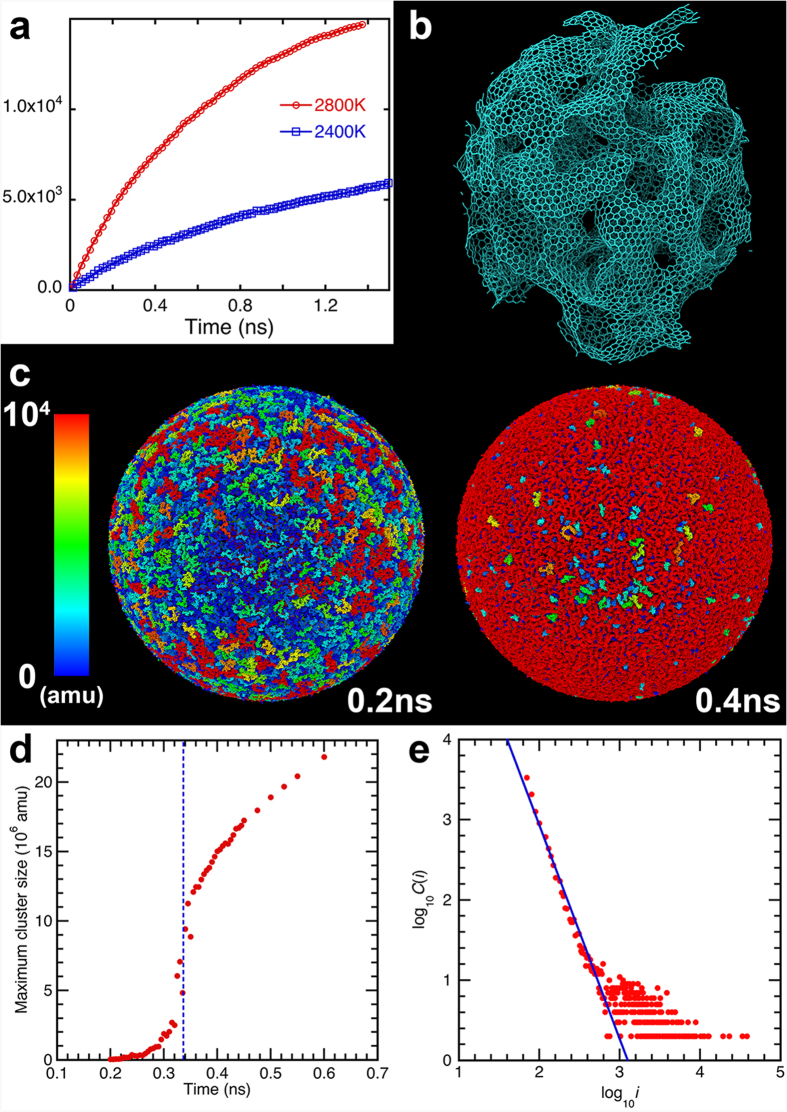
Fractal nanocarbon formed by percolation during nSiC oxidation. (**a**) The time evolution of sp^2^ carbon atoms at temperatures 2,400 K (blue) and 2,800 K (red) for *D* = 10 nm. (**b**) A simulation snapshot at 2 ns shows the structure of the nanocarbon synthesized by oxidation of nSiC (*D* = 10 nm) at 2,800 K. (**c**) Graphene-like carbon clusters produced on the surface of an oxidizing nSiC of diameter *D* = 100 nm at time 0.2 and 0.4 ns, exhibiting a percolation transition. The color represents the cluster mass in atomic mass unit (amu). (**d**) The size of the largest carbon cluster in amu as a function of time (*D* = 100 nm), where the blue dashed line marks the percolation transition. (**e**) Number of clusters, *C*(*i*), as a function of the cluster size, *i*, just before the percolation transition (0.3 ns) for *D* = 100 nm. The blue line shows the power-law fit for larger clusters, *C*(*i*) > 10.

**Figure 3:**
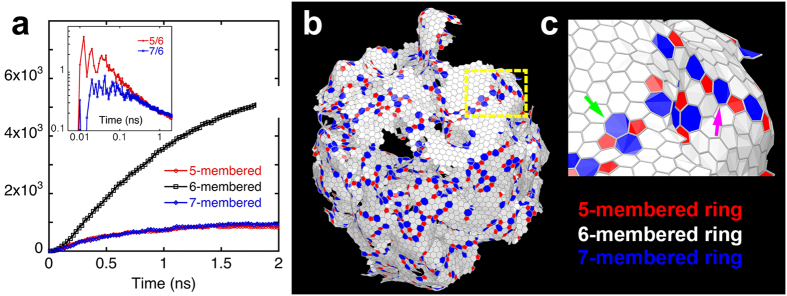
Topology of nanocarbon product. (**a**) Time evolution of 5-, 6-, and 7-membered carbon rings. Inset shows the populations of 5- and 7-membered rings normalized by the number of 6-membered rings for *D* = 10 nm. (**b**) Spatial distributions of 5-membered (red) and the 7-membered (blue) rings superimposed on 6-membered rings (white) at 1.37 ns. (**c**) Close-up view of the area enclosed with the yellow-dotted line in (**b**). The magenta arrow points to alternating 5-membered and 7-membered rings forming a grain boundary, and the green arrow indicates a Stone-Wales defect.

**Figure 4:**
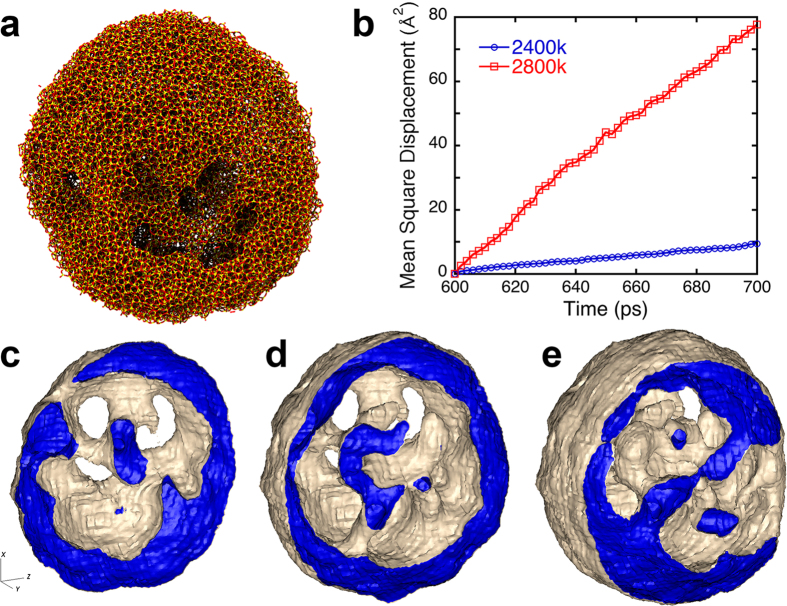
Multifunctional porous silica shell as a nanocapsule and nanoreactor. (**a**) Snapshot of the silica nanocapsule in a 10 nm nSiC at 2,800 K after 2 ns. Here yellow and red spheres are silicon and oxygen atoms, respectively. (**b**) Mean square displacement of oxygen atoms covalently bonded to silicon atoms. At 2,800 K, oxygen atoms diffuse rapidly through the silica shell, facilitating oxidation reactions at the interface of the SiC core. (**c–e**) Morphologies of the silica shell show that silica layer forms a highly porous spherical shell structure. Isosurface plots at mass densities 2.1 g/cm^3^ and 2.2 g/cm^3^ are used to represent the outer surface (gray) and interface (blue) of the silica shell, respectively. Images c to e show *xz* cut surfaces at three different *y* positions.

## References

[b1] Nakamura D. . Ultrahigh-quality silicon carbide single crystals. Nature 430, 1009–1012 (2004). 15329716 10.1038/nature02810

[b2] Eddy C. R. & Gaskill D. K. Silicon carbide as a platform for power electronics. Science 324, 1398–1400 (2009). 19520947 10.1126/science.1168704

[b3] Krenkel W. & Berndt F. C/C-SiC composites for space applications and advanced friction systems. Mater Sci Eng a 412, 177–181 (2005).

[b4] Abrahamson J. & Dinniss J. Ball lightning caused by oxidation of nanoparticle networks from normal lightning strikes on soil. Nature 403, 519–521 (2000). 10676954 10.1038/35000525

[b5] Nakao W. & Abe S. Enhancement of the self-healing ability in oxidation induced self-healing ceramic by modifying the healing agent. Smart Mater Struct 21, 025002 (2012).

[b6] Ohkura Y., Rao P. M. & Zheng X. L. Flash ignition of Al nanoparticles: Mechanism and applications. Combust Flame 158, 2544–2548 (2011).

[b7] White B. . Complete CO oxidation over Cu_2_O nanoparticles supported on silica gel. Nano Letters 6, 2095–2098 (2006). 16968032 10.1021/nl061457v

[b8] Sutter E. A., Tong X., Jungjohann K. & Sutter P. W. Oxidation of nanoscale Au-In alloy particles as a possible route toward stable Au-based catalysts. P Nat Acad Sci USA 110, 10519–10524 (2013). 10.1073/pnas.1305388110 PMC3696783 23754412

[b9] Deal B. E. & Grove A. S. General relationship for thermal oxidation of silicon. J Appl Phys 36, 3770–3778 (1965).

[b10] Opila E. J. Oxidation kinetics of chemically vapor-deposited silicon-carbide in wet oxygen. J Am Ceram Soc 77, 730–736 (1994).

[b11] Newsome D. A., Sengupta D., Foroutan H., Francis Russo M. & van Duin A. C. T. Oxidation of silicon carbide by O_2_ and H_2_O: a ReaxFF reactive molecular dynamics study: Part I. J Phys Chem C 116, 16111–16121 (2012).

[b12] Chroneos A., Yildiz B., Tarancon A., Parfitt D. & Kilner J. A. Oxygen diffusion in solid oxide fuel cell cathode and electrolyte materials: mechanistic insights from atomistic simulations. Energ Environ Sci 4, 2774–2789 (2011).

[b13] Manaa M. R., Reed E. J., Fried L. E. & Goldman N. Nitrogen-rich heterocycles as reactivity retardants in shocked insensitive explosives. J Am Chem Soc 131, 5483–5487 (2009). 19323461 10.1021/ja808196e

[b14] Li Y., Kalia R. K., Nakano A., Nomura K. & Vashishta P. Multistage reaction pathways in detonating high explosives. Appl Phys Lett 105, 204103 (2014).

[b15] Frenklach M., Carmer C. S. & Feigelson E. D. Silicon-carbide and the origin of interstellar carbon grains. Nature 339, 196–198 (1989).

[b16] Chang K. C., Nuhfer N. T., Porter L. M. & Wahab Q. High-carbon concentrations at the silicon dioxide-silicon carbide interface identified by electron energy loss spectroscopy. Appl Phys Lett 77, 2186–2188 (2000).

[b17] Zheleva T. . Transition layers at the SiO_2_/SiC interface. Appl Phys Lett 93, 022108 (2008).

[b18] Shen X. A. & Pantelides S. T. Identification of a major cause of endemically poor mobilities in SiC/SiO_2_ structures. Appl Phys Lett 98, 053507 (2011).

[b19] Badami D. V. Graphitization of alpha-silicon carbide. Nature 193, 569–570 (1962).

[b20] Campbell T. J. . Dynamics of oxidation of aluminum nanoclusters using variable charge molecular-dynamics simulations on parallel computers. Phys Rev Lett 82, 4866–4869 (1999).

[b21] Nakano A., Kalia R. K. & Vashishta P. Growth of pore interfaces and roughness of fracture surfaces in porous silica - million particle molecular-dynamics simulations. Phys Rev Lett 73, 2336–2339 (1994). 10057034 10.1103/PhysRevLett.73.2336

[b22] Nakano A., Bi L. S., Kalia R. K. & Vashishta P. Structural correlations in porous silica - molecular-dynamics simulation on a parallel computer. Phys Rev Lett 71, 85–88 (1993). 10054379 10.1103/PhysRevLett.71.85

[b23] Huang P. Y. . Grains and grain boundaries in single-layer graphene atomic patchwork quilts. Nature 469, 389–392 (2011). 21209615 10.1038/nature09718

[b24] Kroto H. W., Heath J. R., Obrien S. C., Curl R. F. & Smalley R. E. C-60 - Buckminsterfullerene. Nature 318, 162–163 (1985).

[b25] Grantab R., Shenoy V. B. & Ruoff R. S. Anomalous strength characteristics of tilt grain boundaries in graphene. Science 330, 946–948 (2010). 21071664 10.1126/science.1196893

[b26] Yazyev O. V. & Louie S. G. Electronic transport in polycrystalline graphene. Nat Mater 9, 806–809 (2010). 20729847 10.1038/nmat2830

[b27] Stone A. J. & Wales D. J. Theoretical studies of icosahedral C_60_ and some related species. Chem Phys Lett 128, 501–503 (1986).

[b28] Wang Y. . Quantum chemical simulations reveal acetylene-based growth mechanisms in the chemical vapor deposition synthesis of carbon nanotubes. Carbon 72, 22–37 (2014).

[b29] Krishnan A. . Graphitic cones and the nucleation of curved carbon surfaces. Nature 388, 451–454 (1997).

[b30] Nomura K., Chen Y., Kalia R. K., Nakano A. & Vashishta P. Defect migration and recombination in nanoindentation of silica glass. Appl Phys Lett 99, 111906 (2011).

[b31] Wang L. P. . Discovering chemistry with an *ab initio* nanoreactor. Nat Chem 6, 1044–1048 (2014). 25411881 10.1038/nchem.2099 PMC4239668

[b32] Wu C. J., Fried L. E., Yang L. H., Goldman N. & Bastea S. Catalytic behaviour of dense hot water. Nat Chem 1, 57–62 (2009). 21378802 10.1038/nchem.130

[b33] Shimamura K. . Hydrogen-on-demand using metallic alloy nanoparticles in water. Nano Lett 14, 4090–4096 (2014). 24960149 10.1021/nl501612v

[b34] Liu B. L. . Metal-catalyst-free growth of single-walled carbon nanotubes. J Am Chem Soc 131, 2082–2083 (2009). 19170494 10.1021/ja8093907

[b35] Bachmatiuk A. . Investigating the graphitization mechanism of SiO_2_ nanoparticles in chemical vapor deposition. ACS Nano 3, 4098–4104 (2009). 19908851 10.1021/nn9009278

[b36] Takagi D., Hibino H., Suzuki S., Kobayashi Y. & Homma Y. Carbon nanotube growth from semiconductor nanoparticles. Nano Lett 7, 2272–2275 (2007). 17638391 10.1021/nl0708011

[b37] Zhang L. . Porous 3D graphene-based bulk materials with exceptional high surface area and excellent conductivity for supercapacitors. Sci Rep 3, 1408 (2013). 23474952 10.1038/srep01408 PMC3593215

[b38] Xiao J. . Hierarchically porous graphene as a lithium-air battery electrode. Nano Lett 11, 5071–5078 (2011). 21985448 10.1021/nl203332e

[b39] Botsoa J. . Application of 3C-SiC quantum dots for living cell imaging. Appl Phys Lett 92, 173902 (2008).

[b40] Zhao Z. S. . Nanoarchitectured materials composed of fullerene-like spheroids and disordered graphene layers with tunable mechanical properties. Nat Commun 6, 6212 (2015). 25648723 10.1038/ncomms7212

[b41] Paulose J., Chen B. G. G. & Vitelli V. Topological modes bound to dislocations in mechanical metamaterials. Nat Phys 11, 153–156 (2015).

[b42] Berman D., Deshmukh S. A., Sankaranarayanan S. K. R. S., Erdemir A. & Sumant A. V. Macroscale superlubricity enabled by graphene nanoscroll formation. Science 348, 1118–1122 (2015). 25977372 10.1126/science.1262024

[b43] Ajayan P. M. Nanotubes from carbon. Chem Rev 99, 1787–1799 (1999). 11849010 10.1021/cr970102g

[b44] Gogotsi Y. G. & Yoshimura M. Formation of carbon-films on carbides under hydrothermal conditions. Nature 367, 628–630 (1994).

[b45] Ohtomo Y., Kakegawa T., Ishida A., Nagase T. & Rosing M. T. Evidence for biogenic graphite in early Archaean Isua metasedimentary rocks. Nat Geosci 7, 25–28 (2014).

[b46] Lin T. Q. . Nitrogen-doped mesoporous carbon of extraordinary capacitance for electrochemical energy storage. Science 350, 1508–1513 (2015). 26680194 10.1126/science.aab3798

