# Supplementary material for: Nanocarbon synthesis by high-temperature oxidation of nanoparticles
Source: Sci Rep. 2016 Apr 20;6:24109. doi: 10.1038/srep24109 (PMC4837334; doi:10.1038/srep24109)
Supplement: Supplementary Information [file srep24109-s1.pdf]

SUPPLEMENTARY INFORMATION

Nanocarbon synthesis by high-temperature oxidation of nanoparticles

Ken-ichi Nomura,[1] Rajiv K. Kalia,[1] Ying Li,[2] Aiichiro Nakano,[1*] Pankaj Rajak,[1] Chunyang Sheng,[1] Kohei Shimamura,[1,3,4] Fuyuki Shimojo,[3] Priya Vashishta[1]

[1]Collaboratory for Advanced Computing and Simulations, Department of Physics & Astronomy, Department of Computer Science, Department of Chemical Engineering & Materials, and Department of Biological Sciences, University of Southern California, Los Angeles, CA 90089-0242, USA.
[2]Argonne Leadership Computing Facility, Argonne National Laboratory, Argonne, IL 60439, USA.
[3]Department of Physics, Kumamoto University, Kumamoto 860-8555, Japan.
[4]Department of Computational Science, Kobe University, Kobe 657-8501, Japan.
*e-mail: anakano@usc.edu

Section 1. Reactive Force Field (ReaxFF) Molecular Dynamics

In order to study the dynamics of millions of atoms involving chemical reactions, we have developed a scalable parallel implementation of reactive force-field (ReaxFF) molecular dynamics (MD) simulation[1-4] based on spatial decomposition and message passing[5,6]. To describe chemical reactions with moderate computational costs, the first principles-based ReaxFF allows bond breaking and bond formation through reactive bond orders and dynamical charges by employing an electronegativity-equalization scheme. ReaxFF parameters to study high-temperature oxidation of SiC has recently been developed[7,8]. The force fields were thoroughly validated against *ab initio* and experimental results for the equation of state, oxygen binding energy on SiC, heats of formation of major reactants and reaction products, and activation barriers of key chemical reactions[7].

Section 2. Validation by Quantum Molecular Dynamics (QMD) Simulation

To validate the ReaxFF parameters, we performed QMD simulation[9-14] for a small SiC nanoparticle (nSiC) in oxygen environment. The electronic states were calculated using the projector-augmented-wave (PAW) method[15,16], which is an all-electron electronic-structure calculation method within the frozen-core approximation. Projector functions were generated for the 3s, 3p and 3d states of Si, and the 2s and 2p states of C and O. In the framework of density functional theory (DFT), the generalized gradient approximation (GGA)[17] was used for the exchange-correlation energy with nonlinear core corrections[18]. The momentum-space formalism was utilized[19], where the plane-wave cutoff energies were 30 and 250 Ry for the electronic pseudo-wave functions and the pseudo-charge density, respectively. The energy functional was minimized iteratively using a preconditioned CG method[20,21].

We validated the reactive molecular dynamics (RMD) method against QMD simulation for the oxidation of a $Si_{25}C_{25}$ particle immersed in gas of 50 O_2 molecules at 2,800 K. Figure S1, a and b, shows the numbers of Si-O and C-O bonds as a function of time during QMD and RMD

simulations, respectively. In both simulations, much more Si-O bonds are formed than C-O bonds. Also, in both simulations, C-C bonds are formed at the oxidation front, and these C-C bonds nucleate C clusters. Subsequently, these C clusters grow and coalesce into larger C clusters. Figure S1, c and d, shows time evolution of the number of C clusters (blue line) and the size of the largest C cluster (red line) in QMD and RMD simulations, respectively. Both simulations are consistent in showing the condensation of a large number of C clusters into progressively larger C clusters.

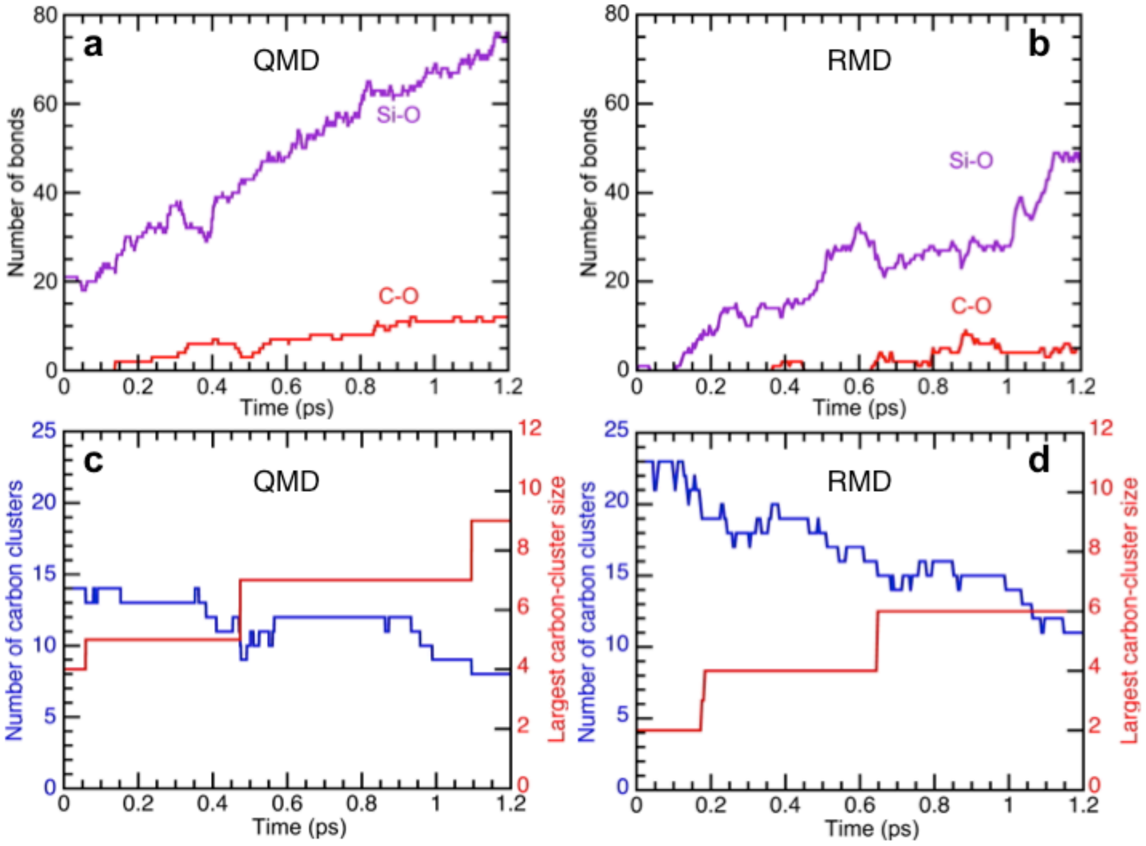

Figure S1 | Validation of RMD against QMD. a, b, The time evolution of the numbers of Si-O (purple) and C-O (red) bonds produced during (a) QMD and (b) RMD simulations, respectively. **c, d**, The time evolution of the number of C clusters (blue) and the size of the largest C cluster (red) in (c) QMD and (d) RMD simulations, respectively.

An O_2 molecule has a spin-triplet ground state, and its reaction with nSiC may be different from that of diamagnetic O_2 in the spin-restricted QMD simulation shown above. In order to study the effect of spin multiplicity, we performed another QMD simulation to account for spin-triplet O_2 molecules. In the new spin-unrestricted QMD simulation, O_2 molecules were randomly assigned either a spin up-up or down-down configuration with equal probability, where the magnetic moment was $|M_s| = 2$ μB/molecule. The simulation schedule was identical to the original diamagnetic QMD simulation explained above. Figure S2, a and b, compares the numbers of Si-O and C-O bonds, respectively, as a function of time during both spin-unrestricted (blue curves) and spin-restricted (red curves) QMD simulations. Figure S2, c and d, shows time evolution of the number of C clusters and the size of the largest C cluster, respectively, in spin-

unrestricted (blue curves) and spin-restricted (red curves) QMD simulations. The results show quantitative agreements between the triplet (or spin-unrestricted) and diamagnetic (or spin-restricted) QMD simulations on key reactivity, *i.e.*, much more Si-O bonds are formed than C-O bonds, while significant production of C-C clusters is observed. The similarity between the reaction of triplet O_2 molecules and that of diamagnetic O_2 molecules with nSiC may be a consequence the high temperature. This, in conjunction with Fig. S1, fully validates our RMD simulations.

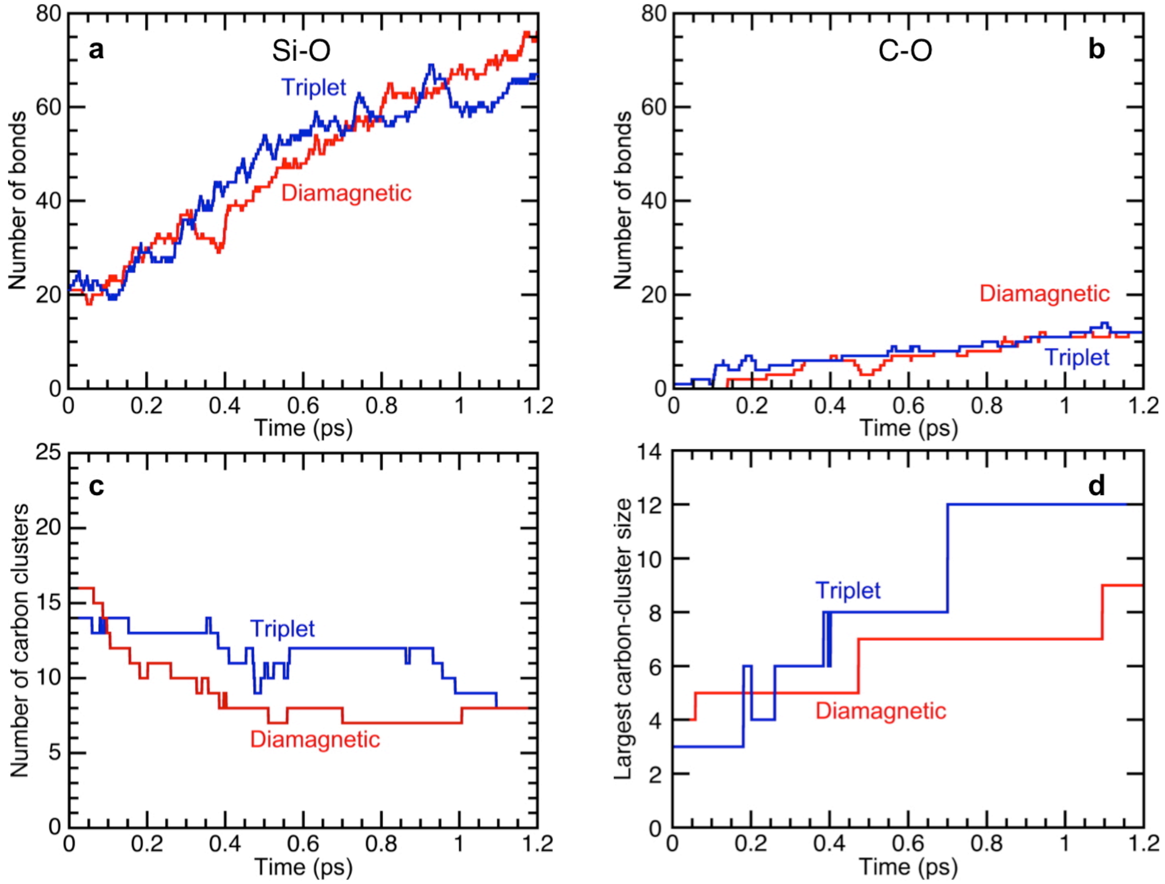

Figure S2 | Effects of spin multiplicity. The time evolution of the number of Si-O bonds (a), number of C-O bonds (b), number of C clusters (c), and size of the largest C cluster produced during spin-unrestricted (blue curves) and spin-restricted (red curves) QMD simulations.

Section 3. Oxidation and Carbon Production Mechanisms

High-temperature oxidation of nSiC: QuickTime movie, S1.mov, shows the oxidation of a 10-nm nSiC at 2,800 K. The movie shows atomic bonds, where silicon, carbon and oxygen atoms are colored in yellow, blue and red, respectively. For the clarity of presentation, O_2 molecules are not shown.

Percolation transition: QuickTime movie, S2.mov, animates the percolation transition for carbon clusters during the oxidation of a 100-nm nSiC at 2,800 K. In the movie, the cluster size is color-coded.

Nanocarbon condensation: Figure S3 shows time evolution of the number of carbon clusters (blue) and the size of the largest carbon cluster (red) for D = 46 nm. The figure shows a

percolation transition at time ~0.2 ns, which is similar to the case of D = 100 nm in the main text.

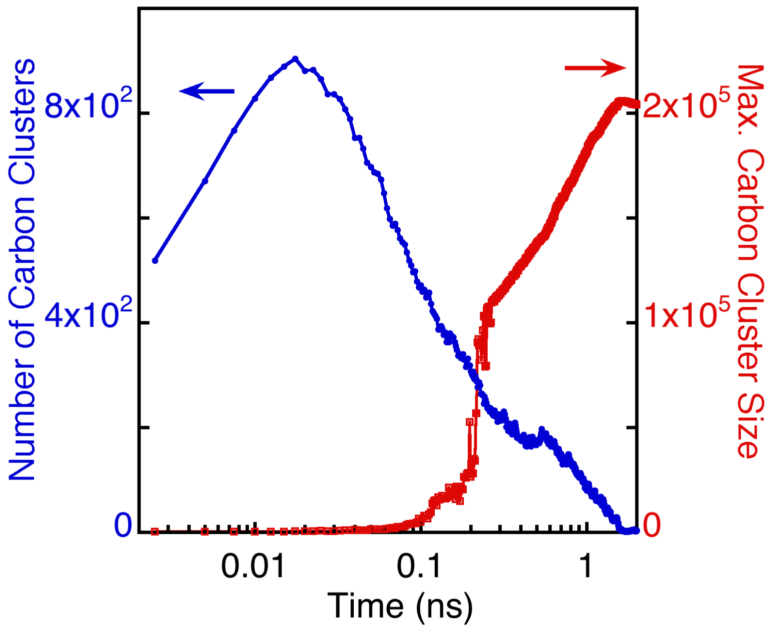

Figure S3 | Percolating nanocarbon. Number of carbon clusters (blue) and the size of the largest carbon cluster (red) in D = 46 nm simulation.

Graphene-sheet with topological defects: QuickTime movie, S3.mov, animates the formation of graphene sheets during the oxidation of a nSiC (D = 10 nm) at 2,800 K. The movie only shows 5-, 6 and 7-membered rings that are colored in red, blue and white, respectively.

Nanocarbon solid: We estimated the mass density of a face-centered cubic (fcc) crystal composed of nanocarbon clusters shown in Fig. 2b. We varied the lattice constant of the crystal, and calculated the energy for each lattice constant by relaxing the atomic positions using the CG method. Figure S4 shows the calculated energy as a function of the mass density. The energy takes a minimum value for a mass density of 0.59 g/cm^3. It should be noted that the fcc crystal achieves the greatest packing fraction of $\pi / 3\sqrt{2}$ ~ 0.74048 among all possible packings of spheres. Instead, random packing is more likely for realistic nanocarbon solids, for which the close packing fraction is 0.634. Accordingly, the upper bound for the mass density of random nanocarbon solid is estimated to be 0.59 (g/cm^3) × 0.634/0.74048 ~ 0.50 g/cm^3.

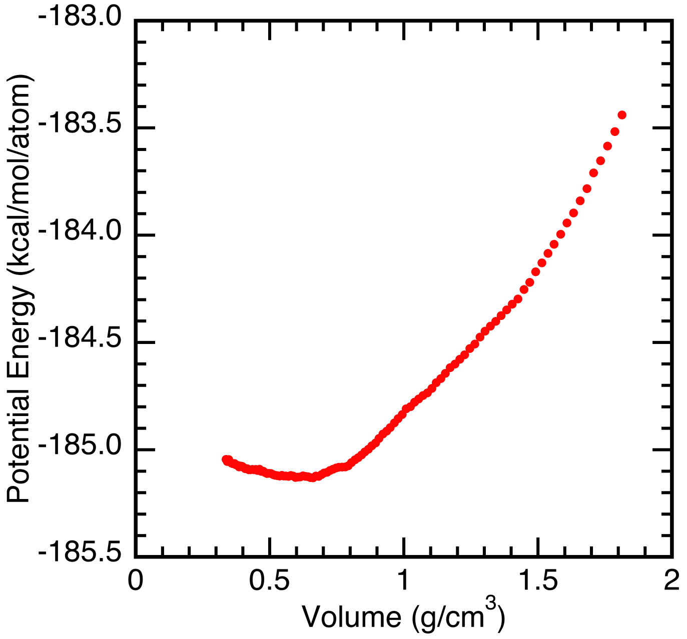

Figure S4 | Mass density of nanocarbon solid. Energy of a face-centered cubic (fcc) solid composed of nanocarbon clusters shown in Fig. 2b.

Mechanical behavior of nanocarbon: QuickTime movie, S4.mov, animates the collision of nanocarbon product in D = 10 nm simulation against a hard wall in the left-hand side. The collision velocity is 1 km/s.

Supplementary References

1 van Duin, A. C. T., Dasgupta, S., Lorant, F. & Goddard, W. A. ReaxFF: a reactive force field for hydrocarbons. *J Phys Chem A* **105**, 9396-9409 (2001).

2 Nomura, K. *et al.* Dynamic transition in the structure of an energetic crystal during chemical reactions at shock front prior to detonation. *Phys Rev Lett* **99**, 148303 (2007).

3 Chen, H. *et al.* Embrittlement of metal by solute segregation-induced amorphization. *Phys Rev Lett* **104**, 155502 (2010).

4 Vedadi, M. *et al.* Structure and dynamics of shock-induced nanobubble collapse in water. *Phys Rev Lett* **105**, 014503 (2010).

5 Nomura, K., Kalia, R. K., Nakano, A. & Vashishta, P. A scalable parallel algorithm for large-scale reactive force-field molecular dynamics simulations. *Comput Phys Commun* **178**, 73-87 (2008).

6 Nomura, K., Small, P. E., Kalia, R. K., Nakano, A. & Vashishta, P. An extended-Lagrangian scheme for charge equilibration in reactive molecular dynamics simulations. *Comput Phys Commun* **192**, 91-96 (2015).

7 Newsome, D. A., Sengupta, D., Foroutan, H., Francis Russo, M. & van Duin, A. C. T. Oxidation of silicon carbide by O_2 and H_2O: a ReaxFF reactive molecular dynamics study, part I. *J Phys Chem C* **116**, 16111-16121 (2012).

8 Newsome, D. A., Sengupta, D. & van Duin, A. C. T. High-temperature oxidation of SiC-based composite: rate constant calculation from ReaxFF MD simulations, part II. *J Phys Chem C* **117**, 5014-5027 (2013).

9 Car, R. & Parrinello, M. Unified approach for molecular-dynamics and density-functional theory. *Phys Rev Lett* **55**, 2471-2474 (1985).

10 Payne, M. C., Teter, M. P., Allan, D. C., Arias, T. A. & Joannopoulos, J. D. Iterative minimization techniques for ab initio total-energy calculations - molecular-dynamics and conjugate gradients. *Rev Mod Phys* **64**, 1045-1097 (1992).

11 Shimojo, F., Ohmura, S., Kalia, R. K., Nakano, A. & Vashishta, P. Molecular dynamics simulations of rapid hydrogen production from water using aluminum clusters as catalyzers. *Phys Rev Lett* **104**, 126102 (2010).

12 Shimamura, K., Shimojo, F., Kalia, R. K., Nakano, A. & Vashishta, P. Bonding and structure of ceramic-ceramic interfaces. *Phys Rev Lett* **111**, 066103 (2013).

13 Shimamura, K. *et al.* Hydrogen-on-demand using metallic alloy nanoparticles in water. *Nano Lett* **14**, 4090-4096 (2014).

14 Shimojo, F. *et al.* A divide-conquer-recombine algorithmic paradigm for multiscale materials modeling. *J Chem Phys* **140**, 18A529 (2014).

15 Blochl, P. E. Projector augmented-wave method. *Phys Rev B* **50**, 17953-17979 (1994).

16 Kresse, G. & Joubert, D. From ultrasoft pseudopotentials to the projector augmented-wave method. *Phys Rev B* **59**, 1758-1775 (1999).

17 Perdew, J. P., Burke, K. & Ernzerhof, M. Generalized gradient approximation made simple. *Phys Rev Lett* **77**, 3865-3868 (1996).

18 Louie, S. G., Froyen, S. & Cohen, M. L. Non-linear ionic pseudopotentials in spin-density-functional calculations. *Phys Rev B* **26**, 1738-1742 (1982).

19 Ihm, J., Zunger, A. & Cohen, M. L. Momentum-space formalism for the total energy of solids. *J Phys C* **12**, 4409-4422 (1979).

20 Kresse, G. & Hafner, J. *Ab-initio* molecular-dynamics simulation of the liquid-metal amorphous-semiconductor transition in germanium. *Phys Rev B* **49**, 14251-14269 (1994).

21 Shimojo, F., Kalia, R. K., Nakano, A. & Vashishta, P. Linear-scaling density-functional-theory calculations of electronic structure based on real-space grids: design, analysis, and scalability test of parallel algorithms. *Comput Phys Commun* **140**, 303-314 (2001).
